# Trends in global and national infertility and factors associated with primary infertile couples in recent middle-aged Chinese

**DOI:** 10.1371/journal.pone.0335926

**Published:** 2025-11-11

**Authors:** Huancheng Tang, Jun Yang, Zeshen Wu, Biao Jiang, Wei Sun, Chaoyang Zhang, Zhi Hu, Qiao Fu, Wei Zhang, Siyu Zhang

**Affiliations:** 1 Department of Urology, Wuhan Third Hospital, Tongren Hospital of Wuhan University, Wuhan, Hubei, China; 2 Department of Urology, Shenzhen Nanshan People’s Hospital (NSPH), Shenzhen, Guangdong, China; 3 Department of Urology, the First Affiliated Hospital, Guangzhou Medical University, Guangzhou, Guangdong, China; 4 Institute of Burns, Wuhan Third Hospital, Tongren Hospital of Wuhan University, Wuhan, Hubei, China; Zhejiang University, CHINA

## Abstract

**Background:**

Infertility poses a significant burden on both global and national scales. However, the epidemiology of primary infertility among reproductive-aged couples in China remains poorly understood. Therefore, this study aimed to investigate the global infertility rate and identify factors associated with primary infertility among middle-aged couples in China.

**Methods:**

Cross-sectional data were derived from the Global Burden of Disease (GBD) study and the Chinese Health and Retirement Longitudinal Study (CHARLS), two extensive databases that examined various disease burdens and associated factors at both global and national levels.

**Results:**

From 1990 to 2019, the global infertility population has shown a steady annual increase. In China, the age-standardized prevalence rate of infertility has remained relatively stable over the past three decades. However, this rate was notably higher than the global age-standardized infertility prevalence rate. Our analysis revealed that the prevalence of primary infertility among middle-aged Chinese couples was approximately 1.7% (947,953/56,892,517). Additionally, we identified anxiety as an associated factor with infertility, highlighting the need for increased public attention to mental health in China.

**Conclusions:**

Infertility continued to be a pressing issue on both global and national levels. This situation warranted widespread attention from Chinese policymakers and healthcare managers. The findings might guide future policy-making and medical interventions in China, with a particular focus on supporting the reproductive needs of middle-aged individuals.

## Introduction

Infertility is defined as an inability to conceive without contraception for more than 1 year. The disease has aroused extensive concerns. Globally, the disease burden has increased by 0.37% and 0.29% during the past three decades for females and males, respectively [1].

In China, the epidemiology of infertility among the reproductive population remains poorly understood, except the the population-based study conducted in 2010 [2]. The study showed that the overall prevalence of infertility was 15.5% [2]. However, due to the long elapsed time and a lack of infertility information at a national level, an urgent need exists to explore the prevalence of infertility, particularly primary infertile couples in middle-aged Chinese, which is lacking in the literature. The reproductive population in China, especially the middle-aged, faces a heavy workload and a large amount of stress to fight and live for their life. An overwhelming burden might pose a negative impact on an individual’s reproductive system, resulting in a childless life because of primary infertility [3–6]. Hence, we tried to investigate the global infertility rate and associated factors in the Chinese middle-aged childless population through both the Global Burden of Disease (GBD) study and the Chinese Health and Retirement Longitudinal Study (CHARLS).

## Materials and methods

The study utilized public databases, and institutional review board (IRB) approval was exempted.

### Databases

The GBD study was conducted by the Institute for Health Metrics and Evaluation (IHME) and was available for non-commercial use (https://www.healthdata.org). The study focused on numerous diseases and injuries around the world and in various countries with different socio-demographic indices (SDI) to calculate measures including prevalence, incidence, disease-adjusted life years (DALYs), years lived with disability (YLDs), and years of life lost (YLLs). To determine SDI, the geometric mean of lag-distributed income, average years of schooling among individuals aged 15 years and above, and total fertility rate is calculated [1]. More information on how these estimates were generated could be accessed in related articles [7–9].

CHARLS aimed to collect representative samples of Chinese middle-aged and elderly people with high quality [10]. A multi-stage cross-sectional survey was applied in 150 counties/districts, each with 3 randomly selected primary sample units (PSUs), resulting in 450 villages/resident committees [11–12]. More information was able to be reached at http://charls.pku.edu.cn.

### Infertility definition

Infertility is mainly classified into two types: primary infertility and secondary infertility. The critical distinction between them is the existence of reproductive history. For the GBD database, total infertile (including primary and secondary) prevalence was initially estimated among surveyed married respondents, and then the surveyed population was proportioned to the overall population. The gender factor was taken into account, and a prediction model was established utilizing DisModMR 2.1, a software implementing the Bayesian meta-regression method to calculate non-fatal outcomes based on sparse and heterogeneous epidemiological data [7–9]. However, in our analysis of the Chinese population (CHARLS data), we were only able to identify potential primary infertility populations in an age subgroup, as a result of survey design. We included participants who were cross-sectioned and had been married. According to the report of the National Bureau of Statistics in 2018 (http://www.stats.gov.cn/zt_18555/ztfx/ggkf40n/202302/t20230209_1902601.html), the mean age of marriage and childbirth in China was 25.7 and 26.8, respectively, from which we assumed that most of the fertile couples had attempted to conceive and not utilized contraception for at least 1 year in their thirties. As CHARLS surveyed and represented the Chinese middle-aged population, we restricted the age to 35–50 years old, a group where most Chinese have been married for more than 1 year and taken the responsibility for fostering children if possible. Infertile couples would experience their life without babies during this period. In other words, we considered the childless population as a proxy for the potential primary infertile population.

### Data extraction

To illustrate the global trend of infertility, we initially downloaded data from the GBD website with various parameters, including location data (global, low SDI, low-middle SDI, middle SDI, high-middle SDI, and high SDI), year data (1990–2019), context data (cause), age data (all ages and age-standardized), metric data (number, percent, and rate), measure data (prevalence, DALYs and YLDs), sex data (male and female) and cause data (male infertility and female infertility). Furthermore, we explored the national trend of age-standardized infertility in China, utilizing corresponding parameters.

To demonstrate infertility prevalence in China, we searched the 2015 CHARLS Wave 3 database and emphasized participants’ age between 35–50, an age group that is usually considered to have been married and attempted to conceive for at least 1 year (http://www.stats.gov.cn/zt_18555/ztfx/ggkf40n/202302/t20230209_1902601.html). Then, we relied on the item “xchildnum: total number of respondent’s children”, “xchildnum_alive: number of respondent’s living children”, “cb051_w3_1: number of respondent’s unrecorded living children”, and the question “whether cross-section sample” to identify the possible childless population. Additionally, we excluded participants who had separated, divorced, and not lived with their spouses for reasons like work (item be001). After all these selections, we were able to identify our targeted primary infertile population.

Moreover, to discover associated factors of primary infertility in China, we also collected demographic background data (birth date, gender, and location), health status, and functioning data (history of hypertension, dyslipidemia and diabetes mellitus, sleeping time, nap time, frequency and length of moderate activities, history of smoke and drink, anxiety and depression), individual income data (income per year), biomarker data (height, weight, and waist circumference), weights data (individual weight with household and individual response adjustment and biomarker weight with household and individual response adjustment), sample information data (interview year), PSU data (province English names, city English names, strata, and PSUs) and blood data (blood weight, fasting triglycerides, fasting high-density lipoprotein cholesterol, fasting low-density lipoprotein cholesterol, fasting total cholesterol, fasting blood glucose, and glycated hemoglobin). Categorical variables were classified as follows: location (main city zone, combination zone between urban and rural areas, and village and other places), hypertension (with and without), dyslipidemia (with and without), diabetes mellitus (with and without), moderate activities (yes and no, do at least 10 minutes continuously during a usual week), smoke (still smoke, and quit or never smoke), drink (drink more than once a month, and quit or never drink or drink a little), anxiety (with and without) and depression (with and without).

### Statistical analysis

All the analysis was completed on Stata SE 16.0 (Stata Corp) and GraphPad Prism 9 (GraphPad Software Inc., San Diego, CA). CHARLS data were derived from a multi-stage cross-sectional study in China, and participants were surveyed according to previous stratified regions (strata = 150) with probability proportional to population size. For each region, 3 randomly selected PSUs were surveyed with probability proportional to population. Hence, we decided to utilize sampling weight, strata, and PSUs parameters to comprehensively delineate infertility prevalence among the Chinese elderly population. We would report the calculated population size in our analysis. To explore associated factors in primary infertile couples, we enrolled individuals with available variables in logistic regression analysis. A logistic regression model based on the design of sampling weight was applied to explore the relationship between infertility and the associated factors. Due to the presence of missing values, we finally were only able to include year, location, hypertension, dyslipidemia, diabetes mellitus, sleeping time, nap time, moderate activities, smoke, drink, anxiety, and depression in the logistic regression model. To avoid correlation among variables, multicollinearity tests were performed to examine their independence from one another. Generally, a variance inflation factor (VIF) less than 10 was considered to be without significant multicollinearity. Continuous variables were shown as mean±standard deviation (SD), and categorical variables were represented as percentages. The results were reported as odds ratio (OR) with 95% confidence interval (95% CI). The two-sided test level α was set to 0.05.

## Results

### Global trend of infertility burden from GBD data

**Fig 1:**
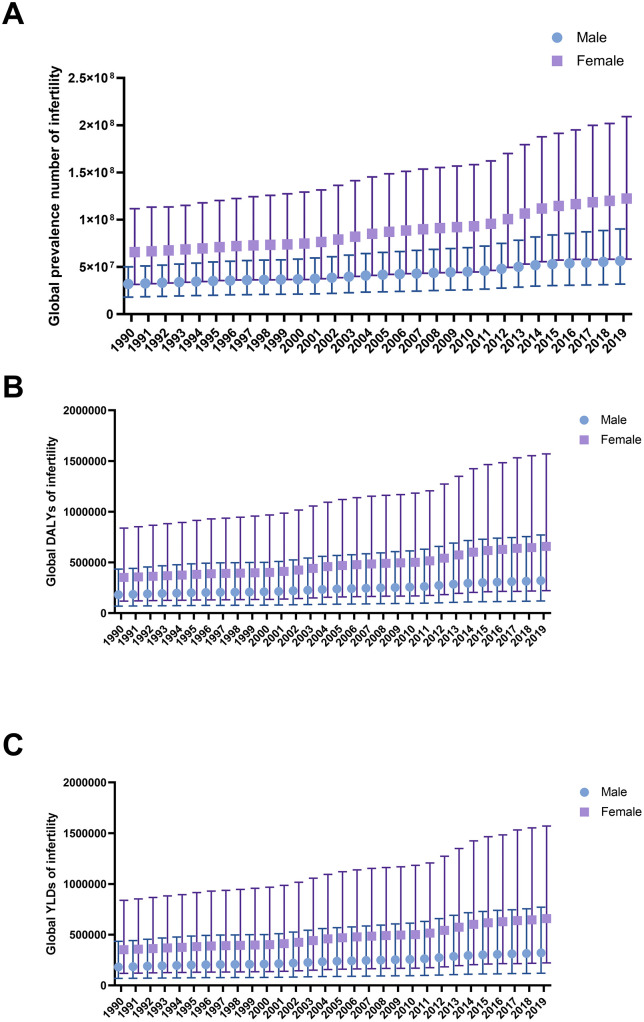
Global trend of infertility from GBD data. **A.** Global prevalence number of infertility. **B.** Global DALYs of infertility. **C.** Global YLDs of infertility. GBD: Global Burden of Disease; DALYs: disease-adjusted life years; YLDs: years lived with disability.

**Fig 2:**
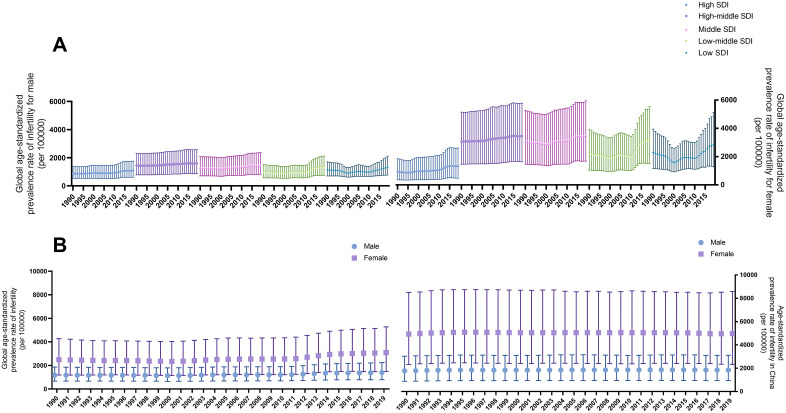
Trends of infertility prevalence around the world and in China (generated from GBD data). **A.** Global age-standardized prevalence rate of infertility among countries with different SDI for male (left) and female (right). **B.** The age-standardized prevalence rate of infertility around the world (left) and in China (right). GBD: Global Burden of Disease; SDI: socio-demographic index.

From 1990 to 2019, the global infertility population has shown a steady annual increase (Fig 1A). The situation was also applied to DALYs and YLDs (Fig 1B, 1C). In the female group, all three parameters were approximately double those in the male group (Fig 1). As for countries falling into different SDI subgroups, we found the prevalence of female infertility in high SDI countries was slightly higher than the prevalence of male infertility after adjusting for age (Fig 2A). While in other subgroups, the discrepancy was more obvious. Both high-middle SDI and middle SDI subgroups had the largest number of infertile people per 100000 population (Fig 2A). In addition, based on the calculated SDI in China, the country has been considered to be among the middle SDI subgroup (S1 Fig), where infertility prevailed and ranked high among subgroups (Fig 2A).

### Prevalence of infertility in China generated from GBD data

In China, the age-standardized prevalence rate of infertility has remained relatively stable over the past three decades (Fig 2B). However, this rate was notably higher than the global age-standardized infertility prevalence rate, particularly for female infertility (Fig 2B).

### Characteristics of the included population in 2015 CHARLS Wave 3

**Fig 3:**
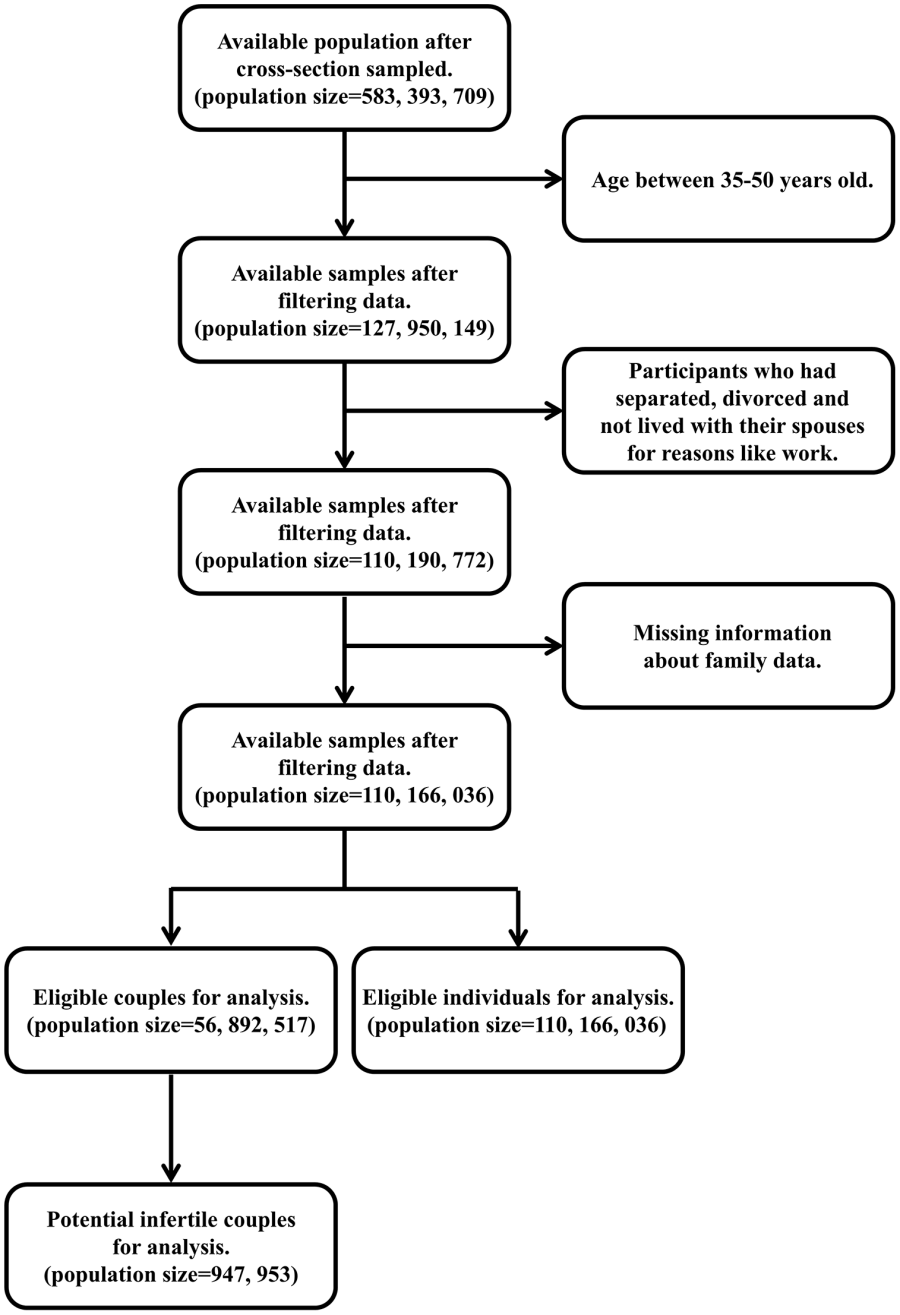
Flow chart of data selection from the CHARLS data. CHARLS: Chinese Health and Retirement Longitudinal Study.

According to the GBD study, we retrieved data from the 2015 CHARLS Wave 3. After filtering ineligible samples, we finally included eligible couples (population size = 56, 892, 517) and individuals (population size = 110, 166, 036) for further analysis, among which we identified 947, 953 potential infertile couples (Fig 3). The rough prevalence rate of primary infertile couples was 1.7% (947, 953/56, 892, 517, Fig 3).

Table 1 demonstrated demographic information, physical examination, history of disease, regular habits, mental health, and individual data. Initially, we found that infertile couples usually had bad life habits, like less moderate activities and drinking a lot. And they also showed psychological disorders, like anxiety and depression (Table 1).

**Table 1:** Characteristics of the eligible Chinese population in CHARLS.

Variables	Infertile couples	Fertile couples	Population size
**Age, yr**	42.48 ± 3.37	47.15 ± 2.44	1137029 vs 109029007
**Location, %**			562192 vs 108740797
** Main city zone**	57.39	21.49	
** Combination zone between urban and rural areas**	9.37	11.02	
** Village and other places**	33.24	67.49	
**BMI, kg/m** ^**2**^	23.17 ± 1.88	24.71 ± 7.26	46791 vs 110466175
**Waist Circumference, cm**	87.67 ± 0.71	85.74 ± 11.86	46791 vs 110229962
**Fasting triglycerides, mg/dl**	101.02 ± 8.70	147.12 ± 97.48	71717 vs 90725744
**Fasting HDL cholesterol, mg/dl**	41.08 ± 4.07	50.23 ± 10.21	71717 vs 90725744
**Fasting LDL cholesterol, mg/dl**	66.15 ± 37.40	99.49 ± 26.28	71717 vs 90725744
**Fasting total cholesterol, mg/dl**	127.83 ± 36.32	180.89 ± 33.65	71717 vs 90725744
**Glycated hemoglobin, %**	5.41 ± 0.49	5.73 ± 0.73	71717 vs 110318840
**Fasting blood glucose, mg/dl**	105.08 ± 3.79	96.62 ± 25.82	71717 vs 91089733
**Hypertension, %**	45.11	19.65	204571 vs 95365863
**Dyslipidemia, %**	0	9.34	267043 vs 106767296
**Diabetes mellitus, %**	0	3.92	267043 vs 107683849
**Sleeping time** ^**#**^ **, h**	7.53 ± 0.56	6.67 ± 1.58	131516 vs 100503997
**Nap time** ^**##**^ **, min**	37.31 ± 45.81	35.58 ± 42.23	166094 vs 100370525
**Moderate activities** ^**###**^ **, %**	14.86	31.61	232729 vs 104893093
**Smoke** ^**####**^ **, %**	22.56	25.53	267043 vs 108500847
**Drink** ^**#####**^ **, %**	32.04	30.06	267043 vs 108443905
**Anxiety** ^**######**^ **, %**	26.12	26.05	166094 vs 100428873
**Depression** ^**######**^ **, %**	10.88	23.99	166094 vs 100558530
**Income** ^**#######**^ **, yuan/yr**	29902.27 ± 26825.17	32124.05 ± 30894.02	73396 vs 41668

CHARLS: Chinese Health and Retirement Longitudinal Study; BMI: body mass index; HDL: high-density lipoprotein; LDL: low-density lipoprotein.

^#^ : Average hours per night during the last month.

^##^ : Average hours for a nap during the last month.

^###^ : Do moderate physical activities that make breathing somewhat harder than normal and may include carrying light loads, bicycling at a regular pace, or mopping the floor. Do at least 10 minutes continuously during a usual week.

^####^ : Defined as used to smoke and have not quit till now.

^#####^ : Defined as used to drink more than once a month and have not quit till now.

^######^ : According to the feelings and behavior during the last week.

^#######^ : According to the last year.

Taking the baseline characteristics into account, we enrolled related factors for further logistic regression analysis. We discovered that after adjusting with several confounders, younger age (OR: 0.68, 95% CI: 0.50–0.92, Table 2), longer nap time (OR: 1.01, 95% CI: 1.00–1.02, Table 2), and presence of anxiety (OR: 9.99, 95% CI: 2.86–34.92, Table 2) were significantly associated with infertile couples. Multicollinearity tests indicated that all included variables in the logistic regression model were far from collinearity (VIF < 10, S1 Table).

**Table 2:** Univariate and multivariate logistic regression analysis of infertile couples.

Variables	Unadjusted OR^*^ (95% CI)	P value	Adjusted OR^*^ (95% CI)	P value
**Year**	0.72 (0.65-0.80)	＜0.001	**0.68 (0.50-0.92)**	**0.012**
**Location**	9.76 (0.61-156.95)	0.107	14.52 (0.35-609.96)	0.160
**Hypertension**	2.36 (0.72-7.68)	0.154	9.98 (0.35-285.71)	0.178
**Dyslipidemia**	NA^**^		NA^**^	
**Diabetes mellitus**	NA^**^		NA^**^	
**Sleeping time** ^**#**^	1.33 (1.19-1.50)	＜0.001	1.46 (0.78-2.72)	0.236
**Nap time** ^**##**^	1.01 (1.00-1.01)	0.027	**1.01 (1.00-1.02)**	**0.030**
**Moderate activities** ^**###**^	NA^**^		NA^**^	
**Smoke** ^**####**^	1.72 (0.53-5.58)	0.366	1.26 (0.18-8.87)	0.812
**Drink** ^**#####**^	6.72 (0.41-109.15)	0.180	17.39 (0.31-971.34)	0.163
**Anxiety** ^**######**^	4.97 (1.55-15.89)	0.007	**9.99 (2.86-34.92)**	**<0.001**
**Depression** ^**######**^	1.18 (0.07-19.21)	0.909	1.02 (0.09-11.81)	0.988

OR: odds ratio; CI: confidence interval.

The data were generated from the CHARLS database.

* : We enrolled individuals with available variables into logistic regression analysis. We finally included year, location, hypertension, dyslipidemia, diabetes mellitus, sleeping time, nap time, moderate activities, smoke, drink, anxiety, and depression into the logistic regression model. In the logistic regression model, the parameters’ codes were as follow: infertile couples (1 = yes, 0 = no); year (continuous variable); location (1= from main city zone, 0 = not from main city zone); hypertension (1 = yes, 0 = no); dyslipidemia (1 = yes, 0 = no); diabetes mellitus (1 = yes, 0 = no); sleeping time (continuous variable); nap time (continuous variable); moderate activities (1 = yes, 0 = no); smoke (1 = yes, 0 = no); drink (1 = yes, 0 = no); anxiety (1 = yes, 0 = no); depression (1 = yes, 0 = no).

** : Not available because individuals with dyslipidemia, diabetes mellitus, and moderate activities were all within fertile couples.

^#^ : Average hours per night during the last month.

^##^ : Average hours for a nap during the last month.

^###^ : Do moderate physical activities that make breathing somewhat harder than normal and may include carrying light loads, bicycling at a regular pace, or mopping the floor. Do at least 10 minutes continuously during a usual week.

^####^ : Defined as used to smoke and have not quit till now.

^#####^ : Defined as used to drink more than once a month and have not quit till now.

^######^ : According to the feelings and behavior during the last week.

## Discussion

Infertility has posed a threat to the global burden of disease and the economy. Several countries and regions have introduced and reported their survey-based or population-based results of infertility prevalence, including Kenya, Japan, the United States, the United Kingdom, Turkey, Iran, Tanzania, New Zealand, and Canada [13–23]. In China, there have also been several studies trying to explore the prevalence of infertility [2,24,25], among which the most recent and famous one was conducted in 2010 involving 18571 couples in 8 provinces [2]. Nevertheless, it had several limitations. Firstly, it only included part of the Chinese population, and some serious mistakes were found when collecting data in Shandong province. Secondly, it was conducted earlier than our study, and the prevalence of infertility might show a different pattern. Thus, we decided to carry out this study, the latest population-based research conducted in 28 provinces of China in 2015, with a focus on primary infertile couples.

We observed that the overall burden of infertility on a global scale was substantial when considering prevalence, DALYs, and YLDs (Fig 1). After adjusting for age, countries with high-middle or middle SDI levels accounted for the largest share of the infertility burden. China, classified within the middle SDI subgroup, faced a significant challenge from infertility, which was of considerable concern to both the government and the global community (Fig 2A). This observation was further corroborated by the results that the age-standardized prevalence of infertility in China was higher than the global average, regardless of gender or year (Fig 2B).

The data from the GBD study delineated the trend of infertility prevalence at the global and national levels. We further intended to find factors associated with primary infertile couples in the Chinese population. The data from 2015 CHARLS Wave 3 indicated that the overall prevalence of primary infertile couples among the 35–50 age group in China was around 1.7%, which was much lower than that reported by Zhou et al (overall prevalence of infertility among women was 15.5%) [2]. We thought one of the reasons might be that the previous study enrolled participants aged 20–49, while our study only included people in the middle-aged group. Moreover, we only included primary infertile couples for further analysis. The middle-aged population served as a main source contributing to the whole society in China. Piles of work prevented them from reproducing the next generation. Considering the problems of the aging population and the increasing number of childless couples in China, the Chinese government recently promoted the three-child policy and encouraged couples to have as many as three babies [26]. From this point of view, our study had significance in estimating primary infertility prevalence in the middle-aged population, which would support policymaking in China to support middle-aged reproductivity.

After adjusting for other confounding factors, we were able to discover that infertile couples tended to experience more anxiety than fertile couples did (OR: 9.99, 95% CI: 2.86–34.92, Table 2). Chinese middle-aged couples faced more pressure from all aspects of life than other age groups because they had to struggle for themselves, stand on their own, and support their parents in such a rapidly developing society. Under these circumstances, they were inevitably destined to experience feelings of anxiety. Nowadays, more and more publications emphasized the impact of psychological disorders, including anxiety, on reproductivity [27–35]. It seemed that the association between them was far more complicated, and psychological intervention might be helpful for unexplained infertility [29–32]. There have been several studies exploring the influence of anxiety on infertile Chinese [27,33,34], reporting that anxiety indeed impacted reproductivity, which might be through reducing semen quality [27]. Our study also supported the relationship with evidence from a population-based study, indicating anxiety might serve as an associated factor in recent Chinese studies. However, we could not infer a causal effect of anxiety on infertility because this was just a cross-sectional study, which meant infertility might also, in turn, influence anxiety.

In the multivariate logistic regression model, we also found that infertile couples seemed to be younger than fertile couples (OR: 0.68, 95% CI: 0.50–0.92, Table 2). We thought the situation might be impacted by China’s actual condition. Those older couples experienced the period before China’s Open and Reform Policy, during which people had lower literacy levels and were encouraged to raise their young generation. While those younger couples lived in a period when China’s economic level was developing rapidly. They had to learn as much as they could to be competitive and excellent. They were more likely to suffer from reproductive diseases.

In addition, we discovered nap time was slightly longer in infertile couples (OR: 1.01, 95% CI: 1.00–1.02, Table 2). However, we could not draw a clear conclusion, as the OR value was approximately equal to ‘1’ and the significant difference was far from clinical importance. Hence, we have not yet considered a relationship between nap time and infertility.

The study had several strengths. First, CHARLS was a multistage cross-sectional study conducted in most provinces of China. The generated data were able to represent the cohort of Chinese middle-aged individuals. Second, this is the most up-to-date study at a national level to delineate the prevalence of primary infertility in the Chinese middle-aged population. Third, we identified anxiety as an associated factor in recent Chinese infertile couples, which could arouse public concern for mental health in middle-aged Chinese, based on the findings from this population-based study.

### Limitations

The study also had some shortcomings. First, we were not able to obtain data about years of cohabitation or the accurate diagnosis of primary infertility, which was a critical factor in defining infertility. To overcome the limitation, we only included participants at the age of 35–50, an age interval that was thought to have been married and attempted to conceive for at least one year in China. Second, the data were generated in 2015 and could not represent the current Chinese population. However, GBD data implied that the prevalence of infertility in China had been stable for several decades. The results of our study might also be applied to the Chinese population nowadays. Third, the CHARLS data did not report details about gynecological information, including the history of endometriosis, pelvic inflammatory disease, abortion, contraception measures, and intercourse frequency. As a solution, we focused on primary infertility and restricted participants to a rigorous standard by which Chinese couples were bound to have a baby through regular sexual life under the influence of Chinese traditional culture. In addition, data loss due to missing values compromised the external validity of the findings. As the data loss in our study was usually missing not at a random (MNAR), in this situation, the imputation method could induce bias. Moreover, imputation was usually time- and resource-consuming and inexplicable, so we considered not using imputation. We still thought the study was valuable in estimating the Chinese middle-aged primary infertility rate. Future research should employ more robust study designs, such as genome-wide analysis, database research, and multi-omics approaches, to enhance the depth and accuracy of our insights in this field [36–40].

## Conclusions and future implications

In summary, infertility continued to be a pressing issue on both global and national levels. The situation should arouse widespread attention among Chinese authorities. In China, the overall prevalence of primary infertile couples among the middle-aged group was around 1.7%. The findings might guide future policy-making and medical interventions in China, to put an emphasis on supporting the reproductive needs of middle-aged individuals.

## Supporting information

S1 Fig SDI change for China from 1990 to 2019 (generated from GBD data). GBD: Global Burden of Disease; SDI: socio-demographic index.
(PDF)

S1 Table Multicollinearity tests to examine the correlation among the included variables in the logistic regression model. (DOCX)
