# Supplementary figures and images for: Trends in global and national infertility and factors associated with primary infertile couples in recent middle-aged Chinese
Source: PLoS One. 2025 Nov 11;20(11):e0335926. doi: 10.1371/journal.pone.0335926 (PMC12604783; doi:10.1371/journal.pone.0335926)

SDI change for China from 1990-2019

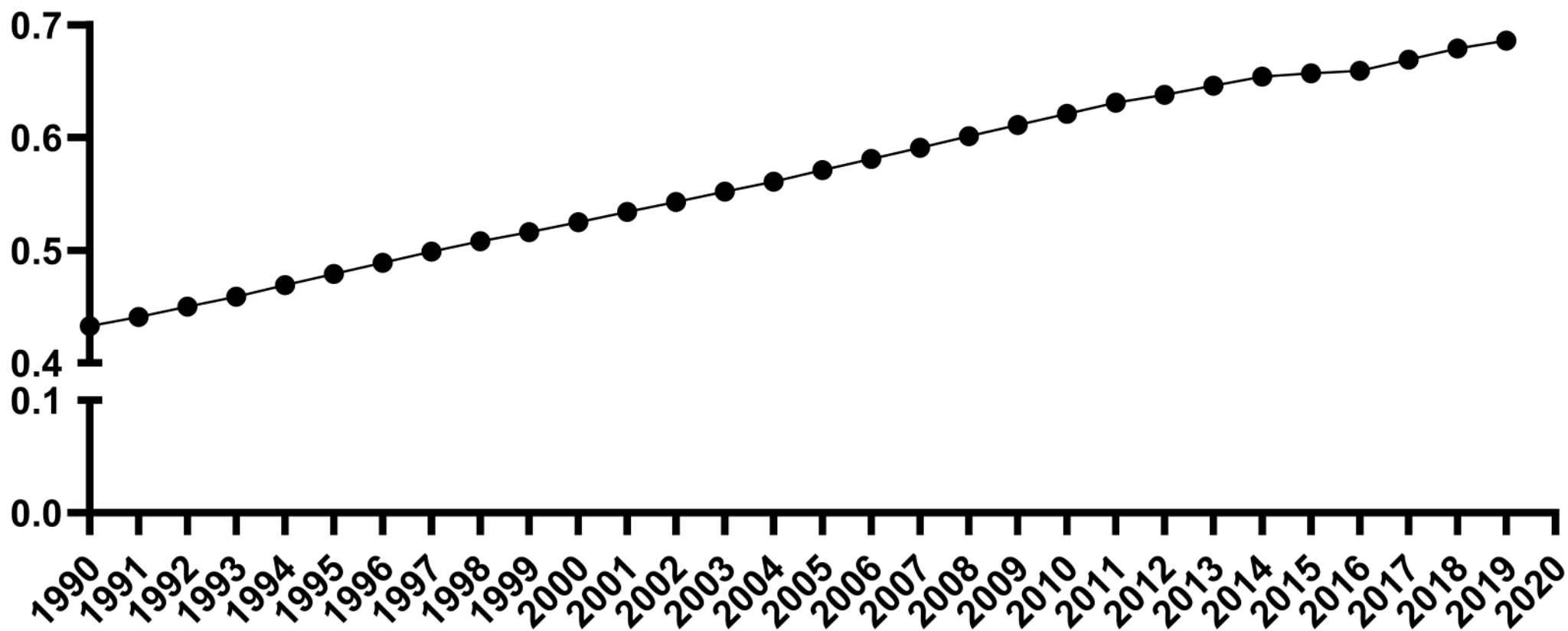

SDI value

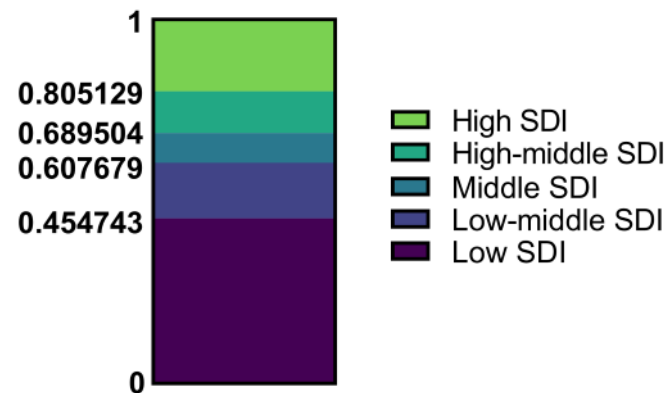

Supplement: S1 Fig — GBD: Global Burden of Disease; SDI: socio-demographic index. (PDF) [file pone.0335926.s001.pdf]
