# Supplementary material for: Trends in global and national infertility and factors associated with primary infertile couples in recent middle-aged Chinese
Source: PLoS One. 2025 Nov 11;20(11):e0335926. doi: 10.1371/journal.pone.0335926 (PMC12604783; doi:10.1371/journal.pone.0335926)
Supplement: S1 Table — (DOCX) [file pone.0335926.s002.docx]

**S1 Table. Multicollinearity tests to examine the correlation among the included variables in the logistic regression model.**

| **Variables** | **VIF** |
| --- | --- |
| Year | 1.04 |
| Location | 1.02 |
| Hypertension | 1.10 |
| Dyslipidemia | 1.13 |
| Diabetes mellitus | 1.07 |
| Sleeping time | 1.03 |
| Nap time | 1.02 |
| Moderate activities | 1.01 |
| Smoke | 1.16 |
| Drink | 1.15 |
| Anxiety | 1.32 |
| Depression | 1.33 |

Generally, VIF less than 10 was considered to be without significant multicollinearity.

VIF: variance inflation factor.
